# Influence of the digital file format on radiographic diagnostic in dentistry: a scoping review

**DOI:** 10.1590/1807-3107bor-2024.vol38.0100

**Published:** 2024-09-30

**Authors:** Murilo MIRANDA-VIANA, Matheus SAMPAIO-OLIVEIRA, Rocharles Cavalcante FONTENELE, Deborah Queiroz FREITAS, Francisco HAITER-NETO

**Affiliations:** (a) Universidade Estadual de Campinas – Unicamp, Piracicaba Dental School, Department of Oral Diagnosis, Oral Radiology, Piracicaba, SP, Brazil.; (b) School of Medicine, KU Leuven and Oral and Maxillofacial Surgery, Department of Imaging and Pathology, Leuven, Belgium.

**Keywords:** Data Compression, Dentistry, Diagnostic Imaging, Biomediccal Technology

## Abstract

Given today’s higher demand for online transmission of radiographic images, clinicians and regulatory agencies should be given the evidence they need to guide them in choosing the best image file format to be adopted. To this end, the present scoping review aims to explore, map, and evaluate the literature, with the object of reporting the influence of image file formats on dental diagnostic tasks by assessing intraoral radiographic images. This scoping review complies with PRISMA-ScR. It was customized to assess the risk of bias of the included studies, and was registered on the Open Science Framework platform. The data extraction protocol was developed based on the PCC acronym. An electronic search was conducted in six databases (Pubmed, Web of Science, Scopus, Embase, Lilacs, Cochrane) in December 2023. Original articles were screened, having observational, diagnostic accuracy, and consisting of in vivo or ex vivo laboratory studies investigating the influence of file formats on different diagnostic tasks in dentistry. Eighteen studies, published between the years 1996 and 2022, were included. The following data were extracted from the selected articles: article title, authors’ citation, publication date, country, diagnostic task, image file formats tested, compression level, and main conclusion. The most widely investigated diagnostic task was caries lesions (n = 10), led by root resorptions (n = 3), root fractures (n = 2), periapical lesions (n = 2), and periodontal disease (n = 1). The most commonly used radiographic techniques were periapical (n = 12) and bitewing (n = 6). The most frequently investigated image file formats were JPEG (all studies) and TIFF (n = 10 studies). BMP, PNG, and JPEG2000 were also included in 7, 3 and 3 studies, respectively. No studies included the DICOM file format. In regard to the subjective assessment of the several dental diagnostic tasks, the studies mostly showed that the influence of the file formats was not significant (n = 10/55.5%). As for the quality assessment of the included papers, more than 70% of the studies featured a low risk of bias. Current evidence on image file formats and dental radiographic diagnosis is reliable. Any image file format can be used without impairing diagnostic accuracy.

## Introduction

Dentistry has experienced a digital revolution in the last decades, and digital radiography has become widely used in oral radiology. Digital receptors feature many advantages, such as time savings, reduced X-ray exposure, and more accessible communication between clinicians.^1^ Another important approach already in evidence today is to export radiographs in different digital file formats (e.g. Digital Imaging and Communications in Medicine- DICOM, Tagged Image File Format- TIFF, Windows Bitmap- BMP, Joint Photographic Experts Group- JPEG, and Portable Network Graphics- PNG).^1^In this case, more or less storage space may be required to archive the radiographs, depending on the adopted digital file format.^2^

Considering the clinical scenario, a smaller radiographic file size may be advantageous to avoid the wasting of virtual storage space, and reduce both image transmission time and money.^3^ This goal can be achieve by using two well-known compression methods: lossless, in which the data are preserved, and lossy, in which some image data are negatively affected, albeit commonly not detected by the human eye. Lossless compression methods are used in the TIFF, BMP, and PNG formats, whereas the lossy compression method uses the JPEG format.^3^

Previous studies have assessed the impact of digital file formats on different dental diagnostic tasks, such as dental caries,^2,4-12^ root resorption,^13-15^ periapical lesions,^16,17^ root fractures,^3,18^ and periodontal disease.^19^ However, the methodologies applied by these investigations vary significantly in regard to the compression levels, samples, and digital radiographic systems tested. Therefore, different results were found according to the study design used by each investigation, thus revealing the need to map and evaluate the existing literature to obtain an overview of the information from published studies. The objective was to summarize the impact of the digital file format on dental diagnosis by using intraoral radiography.

A scoping review is appropriate when conducting research analysis, given the diversity in adopted methodologies, and the existence of divergent findings in the literature. It enables consolidating the literature, combining emerging evidence, and synthesizing it to clarify various aspects, as well as assessing the methodological quality of the studies to ensure the reliability of the results obtained.^20^ These interconnected elements provide the essential foundation for exploring issues beyond the mere concerns for determining the efficacy or experience of interventions, and enable establishing a consensus on the subject at issue. It is important to conduct a scoping review to confirm whether current evidence is sufficient, and to guide the clinicians toward the most appropriate radiographic file format for each diagnostic task. Additionally, reviews of this nature can be an important source of information for future research involving the use of digital radiographic images. Furthermore, they play a fundamental role in standardizing studies that use these images, particularly for researchers who do not have expertise in radiology, or who may have limited knowledge of the subject. Thus, the present study aimed to explore, evaluate, and map the literature to report the influence of image file formats on dental diagnostic tasks by assessing intraoral radiographic images.

## Methods

### Protocol and registration

This scoping review was performed in accordance with the most recent checklist of Preferred Reporting Items for Systematic Reviews and Meta-Analyses (PRISMA) Extension for Scoping Reviews (PRISMA-ScR), and was customized to assess the risk of bias of the included studies.^21,22^ It was registered on the Open Science Framework (OSF) platform under DOI identification number: *https://doi.org/10.17605/OSF.IO/APMW2*.

### Research question

A general literature review was conducted to address the objectives of this study. The data extraction selection protocol was developed based on the PCC acronym (Problem, Concept, and Context), considering the underlying problem, the fundamental principles of digital radiography, and the different contexts involved. Accordingly, the problem (P) was original articles investigating the use of intraoral radiographic images; the concept (C) was the different image file formats, and the context (C) was dental diagnostic tasks.

The research questions for the current review were: “Based on the available literature regarding dental radiographs, do digital image file formats affect diagnostic accuracy in studies using a gold standard?” and “Do digitally stored images in compressed formats impair specific diagnostic tasks in dentistry, in comparison with original images?”

### Eligibility criteria

The scope of the research included original research articles covering observational studies, diagnostic accuracy assessments, and in vivo or ex vivo laboratory investigations assessing the influence of image file formats on subjective diagnostic assessments using radiographic images. The study excluded laboratory studies with animals, review articles (narrative or systematic), letters to the editor, case reports, seminar abstracts, articles without an abstract, guidelines, book chapters, original research that objectively assessed the radiographic image quality and/or used imaging modalities different from intraoral and panoramic radiographs. A summary of the inclusion and exclusion criteria is shown in Table 1.

**Table 1:** Eligibility criteria for study selection.

Variable	Inclusion criteria	Exclusion criteria
Type of study	Original research (observational studies, diagnostic accuracy assessments, and in vivo or ex vivo laboratory investigations) articles	Narrative reviews, systematic reviews, letters to the editor, case reports, seminar abstracts, articles without abstract, guidelines, book chapters, original articles with objective analyses, and original articles using different imaging modalities from intraoral radiographs
Area of interest	Dentistry	Other health areas
X-Ray imaging modalities	Intraoral	Extraoral radiographs and tridimensional examination
Diagnostic tasks	Caries, and endodontic and periodontal diagnostic tasks	Other dental conditions or objective analyses
Image file format	TIFF, BMP, DICOM, PNG, and JPEG (and its variations)	-
Language	No restrictions	-
Date of publication	No restrictions	-
Participants	Radiographs images of ex-vivo or in-vivo participants	Animals

### Information sources and search strategies

In May 2022, electronic searches were conducted in Pubmed, Web of Science, Scopus, Embase, Lilacs, and Cochrane. A search was also conducted in Google Scholar (gray literature), and a manual search of the reference list of the included studies was carried out to locate publications that were not identified electronically. An update of the electronic search was performed in December 2023, and alerts were enabled in all the databases. The search strategies that were applied to each database are summarized in Table 2. The references collected for each database were exported to Mendeley desktop (Manager Library, *version 1.19.8.*, Mendeley, Elsevier), and duplicates were removed.

**Table 2:** Search strategies employed in electronic databases on April 5, 2022. Alerts were enabled in all the databases until submission of the manuscript. The last update was performed on December 2023.

Database	Search strategy
Medline-PubMed https://pubmed.ncbi.nlm.nih.gov/pubmed	(dental digital radiography[Mesh] OR dental digital radiography[TiAb] OR Radiography, Bitewing[Mesh] OR Radiography, Bitewing [TiAb] OR periapical radiographic[TiAb] OR occlusal radiographic[TiAb] OR Scanora[TiAb] OR Digora[TiAb]) AND (Image file format[TiAb] OR TIFF[TiAb] OR Tagged Image File Format[TiAb] OR BMP[TiAb] OR Bitmap[TiAb] OR DICOM[TiAb] OR Digital Imaging and Communications in Medicine [TiAb] OR PNG [TiAb] OR Portable Network Graphic[TiAb] OR JPEG[TiAb] OR Joint Photographic Experts Group[TiAb]) AND (Dental Caries[Mesh] OR Dental Caries[TiAb] OR root resorption[Mesh] OR root resorption[TiAb] OR alveolar bone loss[Mesh] OR alveolar bone loss[TiAb] OR furcation defects[Mesh] OR furcation defects[TiAb] OR Periapical Abscess[Mesh] OR Periapical Abscess[TiAb] OR Periodontal Diseases[Mesh] OR Periodontal Diseases[TiAb] OR periapical lesions[TiAb] OR root fractures[TiAb])
Scopus https://www.scopus.com/home.uri	(INDEXTERMS({dental digital radiography} OR {Radiography, Bitewing}) OR TITLE-ABS-KEY({dental digital radiography} OR {periapical radiographic} OR {occlusal radiographic} OR Scanora OR Digora) AND TITLE-ABS-KEY({Image file format} OR TIFF OR {Tagged Image File Format} OR BMP OR Bitmap OR DICOM OR {Digital Imaging and Communications in Medicine} OR PNG OR {Portable Network Graphic} OR JPEG OR {Joint Photographic Experts Group}) AND INDEXTERMS({Dental Caries} OR {root resorption} OR {alveolar bone loss} OR {furcation defects} OR {Periapical Abscess} OR {Periodontal Diseases} OR TITLE-ABS-KEY({Dental Caries} OR {root resorption} OR {alveolar bone loss} OR {furcation defects} OR {Periapical Abscess} OR {Periodontal Diseases} OR {periapical lesions} OR {root fractures}))
Web of Science www.webofscience.com	TS=(“dental digital radiography” OR “Radiography, Bitewing” OR “periapical radiographic” OR “occlusal radiographic” OR “Scanora” OR “Digora”) AND TS=(“Image file format” OR “TIFF” OR “Tagged Image File Format” OR “BMP” OR “Bitmap” OR “DICOM” OR “Digital Imaging and Communications in Medicine” OR “PNG” OR “Portable Network Graphic” OR “JPEG” OR “Joint Photographic Experts Group”) AND TS=(“Dental Caries” OR “root resorption” OR “alveolar bone loss” OR “furcation defects” OR “Periapical Abscess” OR “Periodontal Diseases” OR “periapical lesions” OR “root fracture”)
Embase https://www.embase.com	#1 ‘dental digital radiography’ OR ‘dental digital radiography’:ti,ab,kw OR ‘tooth radiography’/exp OR ‘tooth radiography’:ti,ab,kw OR ‘periapical radiographic’:ti,ab,kw OR ‘occlusal radiographic’:ti,ab,kw OR ‘imaging software’:ti,ab,kw OR ‘intraoral x ray system’:ti,ab,kw
	AND #2 ‘image file format’:ti,ab,kw OR tiff:ti,ab,kw OR ‘tagged image file format’:ti,ab,kw OR bmp:ti,ab,kw OR bitmap:ti,ab,kw OR (‘digital imaging’:ti,ab,kw AND ‘communications in medicine’:ti,ab,kw) OR png:ti,ab,kw OR ‘portable network graphic’:ti,ab,kw OR jpeg:ti,ab,kw OR ‘joint photographic experts group’:ti,ab,kw
	AND #3 ‘image file format’:ti,ab,kw OR ‘dental caries’/exp OR ‘dental caries’:ti,ab,kw OR ‘tooth disease’/exp OR ‘tooth disease’:ti,ab,kw OR ‘alveolar bone loss’/exp OR ‘alveolar bone loss’:ti,ab,kw OR ‘periapical abscess’/exp OR ‘periapical abscess’:ti,ab,kw OR ‘periodontal disease’/exp OR ‘periodontal disease’:ti,ab,kw OR ‘periapical lesions’:ti,ab,kw OR ‘root fractures’:ti,ab,kw
LILACS https://pesquisa.bvsalud.org/portal/	‘dental digital radiography’/exp OR ‘dental digital radiography’:ab,ti OR ‘Radiography, Bitewing’/exp OR ‘Radiography, Bitewing’:ab,ti OR ‘periapical radiographic’:ab,ti OR ‘occlusal radiographic’:ab,ti OR ‘Scanora’:ab,ti OR ‘Digora’:ab,ti AND ‘Image file format’:ab,ti OR ‘TIFF’:ab,ti OR ‘Tagged Image File Format’:ab,ti OR ‘BMP’:ab,ti OR ‘Bitmap’:ab,ti OR ‘DICOM’:ab,ti OR ‘Digital Imaging and Communications in Medicine’:ab,ti OR ‘PNG’:ab,ti OR ‘Portable Network Graphic’:ab,ti OR ‘JPEG’:ab,ti OR ‘Joint Photographic Experts Group’:ab,ti AND ‘Dental Caries’/exp OR ‘Dental Caries’:ab,ti OR ‘root resorption’/exp OR ‘root resorption’:ab,ti OR ‘alveolar bone loss’/exp OR ‘alveolar bone loss’:ab,ti OR ‘furcation defects’/exp OR ‘furcation defects’:ab,ti OR ‘Periapical Abscess’/exp OR ‘Periapical Abscess’:ab,ti OR ‘Periodontal Diseases’/exp OR ‘Periodontal Diseases’:ab,ti OR ‘periapical lesions’:ab,ti OR ‘root fractures’:ab,ti
Cochrane Library https://www.cochranelibrary.com	ID Search Hits
	#1 MeSH descriptor: [Radiography, Dental, Digital] explode all trees 106
	#2 (dental digital radiography):ti,ab,kw 203
	#3 MeSH descriptor: [Radiography, Bitewing] explode all trees 168
	#4 (Radiography, Bitewing):ti,ab,kw 200
	#5 (periapical radiographic):ti,ab,kw 848
	#6 (occlusal radiographic):ti,ab,kw 258
	#7 (Scanora):ti,ab,kw 7
	#8 (Digora):ti,ab,kw 22
	#9 #1 OR #2 OR #3 OR #4 OR #5 OR #6 OR #7 OR #8 1366
	#10 (Image file format):ti,ab,kw 25
	#11 (TIFF):ti,ab,kw 10
	#12 (Tagged Image File Format):ti,ab,kw 3
	#13 (BMP):ti,ab,kw 508
	#14 (Bitmap):ti,ab,kw 5
	#15 (DICOM):ti,ab,kw 186
	#16 (Digital Imaging and Communications in Medicine):ti,ab,kw 156
	#17 (PNG):ti,ab,kw 80
	#18 (Portable Network Graphic):ti,ab,kw 0
	#19 (JPEG):ti,ab,kw 28
	#20 (Joint Photographic Experts Group):ti,ab,kw 14
	#21 #10 OR #11 OR #12 OR #13 OR #14 OR #15 OR #16 OR #17 OR #18 OR #19 OR #20 881
	#22 MeSH descriptor: [Dental Caries] explode all trees 3496
	#23 (Dental Caries):ti,ab,kw7153
	#24 MeSH descriptor: [Root Resorption] explode all trees 186
	#25 (root resorption):ti,ab,kw 784
	#26 MeSH descriptor: [Alveolar Bone Loss] explode all trees 1566
	#27 (alveolar bone loss):ti,ab,kw 2238
	#28 MeSH descriptor: [Furcation Defects] explode all trees 192
	#29 (furcation defects):ti,ab,kw 369
	#30 MeSH descriptor: [Periapical Abscess] explode all trees 46
	#31 (Periapical Abscess):ti,ab,kw 168
	#32 MeSH descriptor: [Periodontal Diseases] explode all trees 8464
	#33 (Periodontal Diseases):ti,ab,kw 2295
	#34 (periapical lesions):ti,ab,kw 393
	#35 (root fractures):ti,ab,kw 306
	#36 #22 OR #23 OR #24 OR #25 OR #26 OR #27 OR #28 OR #29 OR #30 OR #31 OR #32 OR #33 OR #34 OR #35 17832
	#37 #9 AND #21 AND #36 5
Google Scholar https://scholar.google.com	“Radiography” OR “radiographic exams” OR “radiographic imaging” AND “Image file format” OR “imaging file format” OR “TIFF” OR” JPEG” OR “BMP” OR “DICOM” AND “Caries” OR “periapical lesions” OR “root resorption”

### Selection of sources of evidence

After uploading the studies from the databases into Mendeley software, two calibrated reviewers (MMV and MSO) independently performed the initial screening by reading the title and abstract of the references selected from the electronic search. A calibration session was conducted before initiating this step to confirm the agreement between the examiners. Accordingly, 10% of the included references were selected randomly for the examiners to assess independently, and apply the discussed eligible criteria. An almost perfect agreement was obtained between the examiners (kappa = 1.00), according to Landis and Koch;^23^ the two reviewers assessed all the studies independently using a binary scale (0 – article to be excluded, and 1 – article to be included). The studies that fit the eligibility criteria were selected for the full-text evaluation. Upon reading all the titles and abstracts, the kappa test was performed once again to assess the interexaminer agreement, considering the assessment of the whole sample of references. This agreement between the reviewers was found to be almost perfect (kappa = 1.00), indicating no disagreements.^23^ A second screening was executed by reading the full texts of the initially selected articles.

### Data Items

One of the reviewers (MMV) inputted the data extracted from the selected articles into a Microsoft Excel (version 2302, Microsoft Office, Redmond, USA) spreadsheet. A second reviewer (MSO) double-checked the information independently. The following data were extracted from the selected articles: article title, authors’ citation, publication date, country, diagnostic task, radiographic modality, radiographic receptor, phantom, X-ray unit, digital radiographic system, image file formats tested, compression level, number of evaluators, and main conclusion. Discrepancies during the data extraction process were discussed by the two reviewers until a consensus was achieved. In cases where mutual agreement was not reached, a third reviewer was consulted to resolve the impasse. The information on the data extracted from the selected articles is shown in Table 3.

**Table 3:** Data items from articles included in the Scoping Review .

Article Title	Authors’ abbreviated citation	Publication data	Country	Assessed Diagnostic Task	Radiographic technique	Radiographic receptor operated	Phantom used	X-ray unit and system employed	Image file formats evaluated	Compression level/Compression ratio	Number of evaluators	Main conclusion
Impact of lossy image compression on accuracy of caries detection in digital images taken with a storage phosphor system	Wenzel et al.	1996	Denmark	Caries lesions	Periapical	Phosphor Storage Plate (PSP)	116 extracted human premolars and molars	System: Digora (Soredex Medical Systems, Helsinki, Finland)	TIFF and JPEG	1:2, 1:5, 1:12, 1:20, and 1:33	5	Compression ratios higher than 1:12 significantly impair the accuracy of the diagnosis of caries lesions and image quality due to reduction of diagnostic values.
Effect of noise on the compressibility and diagnostic accuracy for caries detection of digital bitewing radiographs	Janhom et al.	1999	Netherlands	Caries lesions	Bitewing	Film-based and Phosphor Storage Plate (PSP)	66 bitewing radiographs of upper and lower pre-molars and molars in occlusion contact	System: Digora® (Soredex Corporation, Helsinki, Finland)	BMP and JPEG	1:1, 1:3, 1:14, 1:21, and 1:34	7	Compression ratios higher than 1:14 significantly impair the accuracy of the diagnosis of caries lesions and image quality due to reduction of diagnostic values.
Interaction between noise and file compression and its effect on the recognition of caries in digital imaging	Janhom et al.	2000	Netherlands	Caries lesions	Bitewing	Film-based and Phosphor Storage Plate (PSP)	59 bitewing radiographs of upper and lower pre-molars and molars in occlusion contact	System: Digora® (Soredex Corporation, Helsinki, Finland)	BMP and JPEG	1:14 and 1:21	7	Compression ratios 1:21 may impair the evaluation and diagnosis of incipient caries lesions due to higher observer error.
Impact of lossy compression on diagnostic accuracy of radiographs for periapical lesions	Eraso et al.	2002	United States of America	Periapical lesions	Periapical	Digital sensor: Charge-coupled device (CCD)	Database - 50 digital radiographs containing single-rooted teeth	System: Schick (Technologies Inc, Long Island, NY) X-ray Unit: Heliodent DS (Sirona, Bensheim, Germany)	JPEG	1, 2, 4, 8, 16, 32, 48, and 64	4	Compression ratios higher than 1:16 can have a severe impact on the diagnosis of periapical lesions due to reduction in diagnostic values.
A comparison of two compression algorithms and the detection of caries	Janhom et al.	2002	Netherlands	Caries lesions	Bitewing	Film-based	100 extracted posterior teeth (premolars and molars) mounted on a plaster block	System: Heliodent MD (Siemens, Bensheim, Germany) Scan unit: Agfa DuoScan T1200 (Agfa, Mortsel, Belgium)	BMP, JPEG, and wavelet	1:1 and 1:9 ;	9	No difference was found between the file formats in the diagnosis of enamel caries lesions. However, JPEG-compressed images performed worse than the original and wavelet-compressed images in detecting dentinal lesions due to higher observer error.
Effect of data compression on proximal caries detection: observer performance with DenOptix® photostimulable phosphor images	Pabla et al.	2003	Brazil	Caries lesions	Periapical	Phosphor Storage Plate (PSP)	41 extracted human posterior teeth (22 molars and 19 premolars, half maxillary and half mandibular)	System: DenOptix (Gendex Dental Systems, Milan, Italy) X-ray Unit: Prostyle (Planmeca Ou, Helsinki, Finland)	JPEG and TIFF	1:2, 1:11, and 1:16	8	The file format of periapical radiographs does not influence the diagnosis of caries lesions.
Effects of JPEG compression in quantitative digital radiographic subtraction of simulated bone loss	Mahl et al.	2003	Brazil	Periodontal disease (Bone loss)	Periapical	Film-based	12 periapical radiographs of the lower molar region	Scan Unit: Perfection 2450® scanner (Epson, USA)	JPEG	1:1, 1:2, 1:3, 1:4, and 1:6	1	Compression levels 8 and 6 overestimated bone loss.
Comparison of JPEG and wavelet compression on intraoral digital radiographic images	Kim, E.	2004	South Korea	Caries lesions	Periapical	Digital sensor: Charge-coupled device (CCD)	30 extracted sound posterior teeth and 30 extracted posterior teeth with occlusal caries mounted on a plaster block	System: Schick (Schick Inc., Long Island, USA),	JPEG and JPEG2000	1:5, 1:9, 1:14, and 1:28	3	Compression rates up to 1:9 for JPEG and 1:14 for JPEG 2000 did not impair caries lesion diagnosis.
The impact of image compression on diagnostic quality of digital images for detection of chemically-induced periapical lesions	Koenig et al.	2004	United States of America	Periapical lesions	Periapical	Digital sensor: Complementary metal–oxide semiconductor (CMOS)	13 human dry mandibles with single- and/or multiple-root teeth inserted in acrylic blocks	System: DX-CS1 (R.C. Eggleton, Consulting, Indianapolis, IN) X-ray Unit: CCX Digital Computer Controlled X-Ray Timer (Trophy Radiologie, Vincennes, France)	JPEG	1:2, 1:14, 1:23, 1:28, and 1:47	3	JPEG compression does not affect the detectability of periapical lesions up to a compression ratio of 1:28.
Reproducibility of and file format effect on digital subtraction radiography of simulated external root resorptions	Gegler et al.	2006	Brazil	External root resorptions	Periapical	Film-based	11 human upper incisors	System: Spectro 70 (Dabi Atlante, Ribeirão Preto, SP, Brazil) Scan unit: Epson Perfection 2450w scanner (Epson, USA)	TIFF, BMP, and JPEG	01:03	3	No difference was found between TIFF, BMP, and JPEG in ERR diagnosis.
Evaluation of JPEG compression on the diagnosis of caries in digitalized radiographs	Bissol et al.	2006	Brazil	Caries lesions	Bitewing	Film-based	20 bitewing radiographs of upper and lower pre-molars and molars in occlusion contact	Scan unit: HP Scanjet 4C	TIFF and JPEG	13 levels of compression	5	Images compressed above or equal to level 9 were acceptable, since they did not impair caries diagnosis. On the other hand, images compressed below or equal to 3 were unacceptable, because they impaired caries diagnosis.
Effect of image compression on the radiographic diagnosis of external root resorptions	Fontanella et al.	2007	Brazil	External root resorptions	Periapical	Film-based	33 upper central incisors inserted in a dry skull	X-ray Unit: Spectro 70 (Dabi Atlante, Brazil) Scan Unit: Perfection 2450® scanner (Epson, USA)	JPEG	1:1, 1:2, 1:6, and 1:7	6	The file format for periapical radiographs does not influence ERR diagnosis.
The effect of wavelet and discrete cosine transform compression of digital radiographs on the detection of subtle proximal caries	Schulze et al.	2008	Germany	Caries lesions	Periapical	Digital sensor: Charge-coupled device (CCD)	51 healthy teeth and 49 teeth with non-cavitated carious lesions at proximal surfaces (33 incisors, 10 canines, 13 premolars and 44 molars). The teeth were inserted in plaster cubes, in pairs, according to their respective dental groups (incisors, premolars, molars)	System: Sirona (Dental Systems, Bensheim, Germany) X-ray Unit: Heliodent DS (Dentsply Sirona, Bensheim, Germany)	TIFF, JPEG and JPEG2000	1:1, and 1:12	10	The file format for periapical radiographs does not influence caries lesion diagnosis.
Evaluation of proximal caries in images resulting from different modes of radiographic digitalization	Xavier et al.	2011	Brazil	Caries lesions	Bitewing	Film-based	56 human posterior teeth (28 premolars and 28 molars)	X-ray Unit: Kaycor X-707 (Yoshida Dental Manufacturing Co., Tokyo, Japan) Scans Units: CanonScan D646U (Canon USA Inc., Newport News, VA) and Genius ColorPage HR7X (KYE Systems Corp. America, Doral, FL)	TIFF and JPEG	1:1, and 1:12	3	The carious lesion diagnosis did not change in either of the file formats (JPEG and TIFF).
Effect of JPEG compression on the diagnostic accuracy of periapical images in the detection of root fracture	Noujeim et al.	2012	United States of America	Root fractures	Periapical	Digital sensor: Complementary metal–oxide semiconductor (CMOS)	10 human dry mandibles containing 151 upper and lower teeth (incisors, canines, premolars, and molars)	System: Suni (Suni Medical Imaging Inc., CA, USA) X-ray Unit: Prostyle (Planmeca Ou, Helsinki, Finland)	JPEG and TIFF	0, 1:4, and 1:18	4	The file format of periapical radiographs does not influence the diagnosis of RF.
Influence of the image file format of digital periapical radiographs on the diagnosis of external and internal root resorptions	Miranda-Viana et al.	2021	Brazil	External and internal root resorptions	Periapical	Phosphor Storage Plate (PSP) Digital sensor: Complementary metal–oxide semiconductor (CMOS)	34 single-rooted human teeth inserted into human dry mandibles	System: Digora Toto (Soredex, Tuusula, Finland) X-ray Unit: Focus (Instrumentarium, Tuusula, Finland)	TIFF, PNG, BMP, and JPEG	0, 1:1, 1:6, and 1:23	5	The file format of periapical radiographs does not influence the diagnosis of IRR or ERR.
Influence of the file format and transmission app on the radiographic diagnosis of caries lesions	Madlum et al.	2021	Brazil	Caries lesions	Bitewing	Digital sensor: Complementary metal–oxide semiconductor (CMOS)	40 human posterior teeth (premolars and molars) inserted in plaster blocks	System: Digora Toto (Soredex, Tuusula, Finland) X-ray Unit: Focus (Instrumentarium, Tuusula, Finland)	TIFF, PNG, BMP, and JPEG	0, 1:1, 1:5, and 1:24	5	The digital file format does not affect the diagnosis of proximal caries lesions.
Digital file format does not influence the radiographic diagnosis of vertical root fracture	Miranda-Viana et al.	2022	Brazil	Vertical root fracture	Periapical	Phosphor Storage Plate (PSP) Digital sensor: Complementary metal–oxide semiconductor (CMOS)	34 single-rooted human teeth, including lower incisors, canines and premolars inserted into human dry mandibles	Systems: Digora Toto (Soredex, Tuusula, Finland) and Digora® Optime (Soredex Corporation, Helsinki, Finland) X-ray Unit: Focus (Instrumentarium, Tuusula, Finland)	TIFF, PNG, BMP, and JPEG	Digora Toto: 0, 1:1, 1:6, and 1:26 Digora Optime: 0, 1:1, 1:6, and 1:20	5	The file format of periapical radiographs does not influence the diagnosis of VRF.

### Quality assessment

The Quality Assessment of Diagnostic Accuracy-2 (QUADAS-2) was applied to the articles included in the final analysis to judge the risk of bias.^24^ Although the risk of bias analysis is not included in the PRISMA-ScR, it was performed to assess the reliability of the results of the articles included in this scoping review. The QUADAS-2 instrument (University of Bristol Resource, Bristol, UK) is composed of four domains: patient selection (D1), index test (D2), reference standard (D3), and flow and timing (D4). These domains were evaluated in two categories (risk of bias and applicability concerns) by two authors (MMV and RCF) through consensus. In cases of disagreement, a third reviewer (FHN) was consulted to achieve consensus. Concerning the risk of bias, each topic (D1, D2, D3, and D4) had to be answered and scored as low risk ‘+’ (positive answers), some concerns ‘–’ (missing information), or high risk ‘x’ (negative answers). Only domains D1, D2 and D3 were scored for applicability concerns. If there was any concern about avoiding the research topic in any of these domains, the risk of bias was considered either high risk ‘x’, or low risk ‘+’ otherwise. If any information was missing, some concerns were applied ‘-’. The overall score was determined based on the scores attributed to the four domains: if all the domains were scored as a low risk of bias ‘+’, the overall score was also judged as low risk ‘+’. However, if one of the domains was considered as having some concerns ‘-’ or high risk of bias, the overall score attributed was the worst possible.

### Synthesis of results

In the scoping review, we extracted key information from the articles in order to address the central question, covering diagnostic tasks, tested image file formats, compression levels, and main conclusions. The results were then grouped and presented visually (see the figures). The scoping review revealed a prevalence of the periapical radiographic technique, focusing on the diagnosis of caries lesions and root resorption. Direct digital sensors were commonly used, and JPEG and TIFF file formats were frequently explored. Most studies concluded that various image file formats did not significantly affect diagnostic accuracy.

## Results

### Study Selection

**Figure 1:**
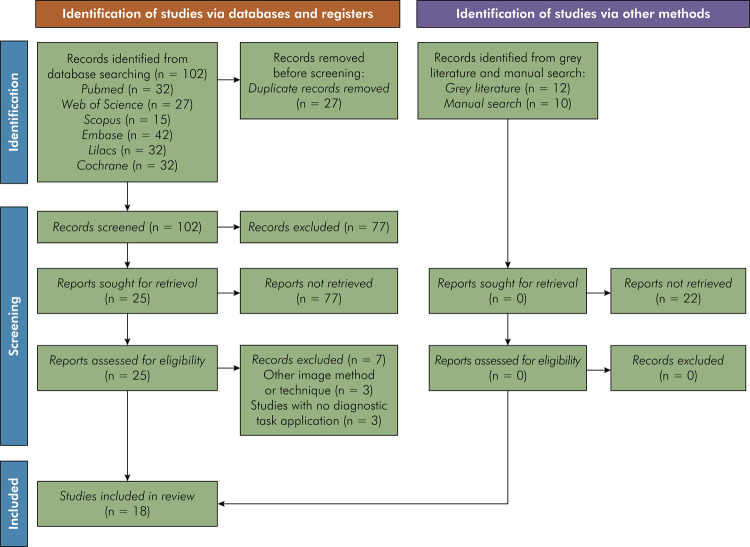
PRISMA 2020 flow diagram including searches of databases, registrations, and other sources.

The electronic search identified 129 studies (Pubmed = 32, Web of Science = 27, Scopus =15, Embase = 42, Lilacs = 8, and Cochrane = 5). No studies from the searches performed in the gray literature or reference lists of the included studies were added. After removing 27 duplicates, 102 studies were selected. In the first screening, 25 titles and abstracts were selected for full-text reading after applying the pre-established eligibility criteria. Seven of these studies were excluded because they did not fit the eligibility criteria adequately, or used another imaging method and/or radiographic technique, or did not include a dental diagnostic task in the main study objective. Thus, at the end of the article selection stage, 18 studies were included in this scoping review to assess the quality of the studies and the risk of bias process. The flowchart of the study selection is shown in Figure 1.

### Study characteristics

**Figure 2:**
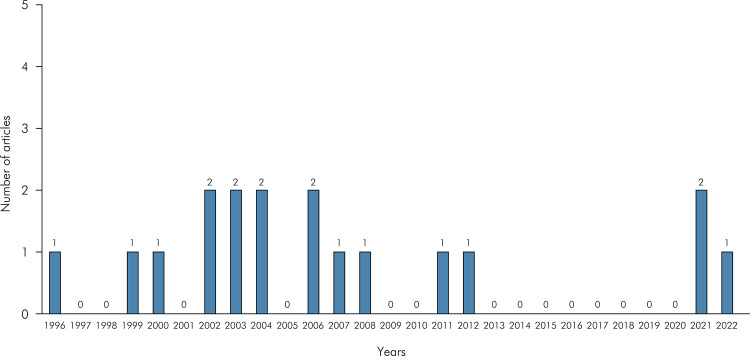
Bar chart showing the number of publications over the years

As seen in Table 3, the first article evaluated among the selected studies to determine the influence of different image file formats on a dental diagnostic task (caries lesions) was published in 1996.^4^ There has been a progressive rise in the number of studies on this subject published over the years, with most studies published between 2002 and 2006. The most recent article was published in 2022 by Miranda-Viana et al.,^3^ and addressed the influence of different image file formats on the diagnosis of root fracture. Figure 2 presents a bar chart with the timeline and number of articles selected for this scoping review.

Regarding the dental diagnostic task assessed in each study, ten assessed caries lesions,^2,4-12^ two assessed periapical lesions,^16,17^ three assessed root resorptions,^13-15^ two assessed root fractures,^3,18^ and one assessed periodontal disease (bone loss).^19^ Most of the studies were based on a methodological design with human teeth inserted in acrylic blocks and/or human dry mandibles.^2-9,10-15,17-19^ Only two studies were based on a retrospective assessment of radiographic images from a clinical database.^10,16^

As for the intraoral radiographic technique used, twelve studies used periapical radiography,^3,4,8,9,11,13-19^and six studies used bitewing radiography.^2,5-7,10,12^ Regarding the types of receptors employed, eight studies used digital sensors,^2,3,9,11,15,16-18^ three used a charge-coupled device (CCD),^9,11,16^ five used a complementary metal–oxide semiconductor (CMOS),^2,3,15,17,18^ six used a photostimulable phosphor plate (PSP),^3-6,8,15^ and six used a film-based scanner and digital printer.^7,10,12-14,19^

**Figure 3:**
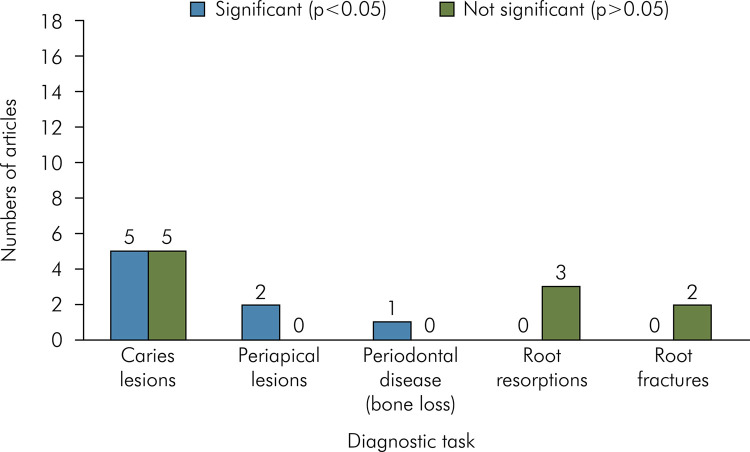
Bar chart displaying the number of studies, according to the diagnostic task, and whether the different file formats influenced it

Regarding the image file format tested, all the studies included JPEG,^2-19^ ten included TIFF,^2-4, 8, 10-13, 18^ seven included BMP,^2-7,13^ three included PNG,^2-4^ and three included JPEG2000, also referred to as the wavelet format.^7,9,11^ As seen in Figure 3 regarding the significant influence of the different image file formats on the diagnostic tasks, eight studies^4-6,9,10,16,17,19^found a significant effect, and ten studies^2,3,7,8,11-15,18^showed no significant influence of the file formats on the subjective assessment of the several dental diagnostic tasks. Among those that showed a significant effect of image compression on the radiographic diagnosis, five assessed caries lesions,^4,5,6,9,10^ two assessed periapical lesions,^16,17^ and one assessed periodontal disease (bone loss).^19^ JPEG file format had the worst diagnostic accuracy across all the studies. Notably, the compression levels that led to reduced accuracy were very high, between 1:30 and 1:47. This is not clinically applicable, because of the huge amount of loss of graphical information from the image; hence, the accuracy of the diagnostic is expected to be reduced.

**Figure 4:**
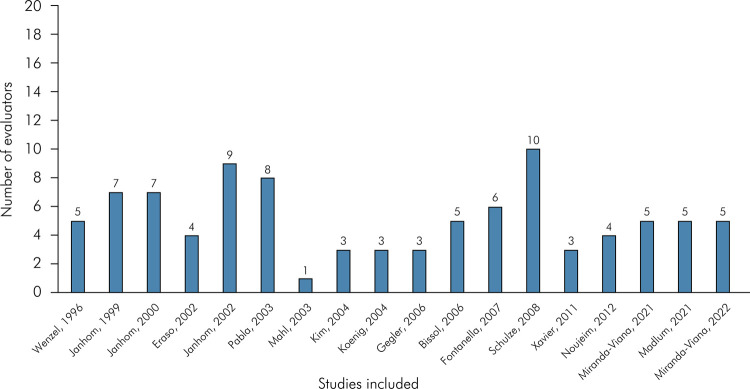
Bar chart indicating the number of evaluators in the included articles

**Figure 5:**
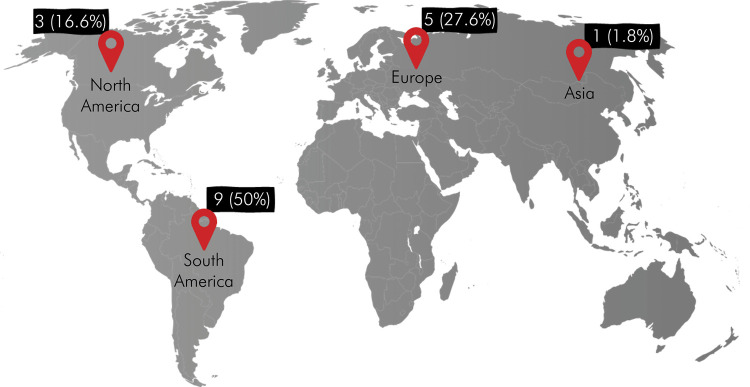
Absolute numbers (percentage) of the articles included in the scoping review, according to geographic location

Regarding the number of evaluators, Figure 4 shows a bar graph for the 18 studies included. The number of evaluators ranged from one to ten, with median (MD), minimum and maximum values of 5, 1, and 10, respectively. Concerning the continent where the studies were published (Figure 5), three were published in North America,^16-18^ nine in South America,^2,3,8,10,12-15,19^ five in Europe^4-7,11^and one in Asia.^9^

### Quality assessment

**Figure 6:**
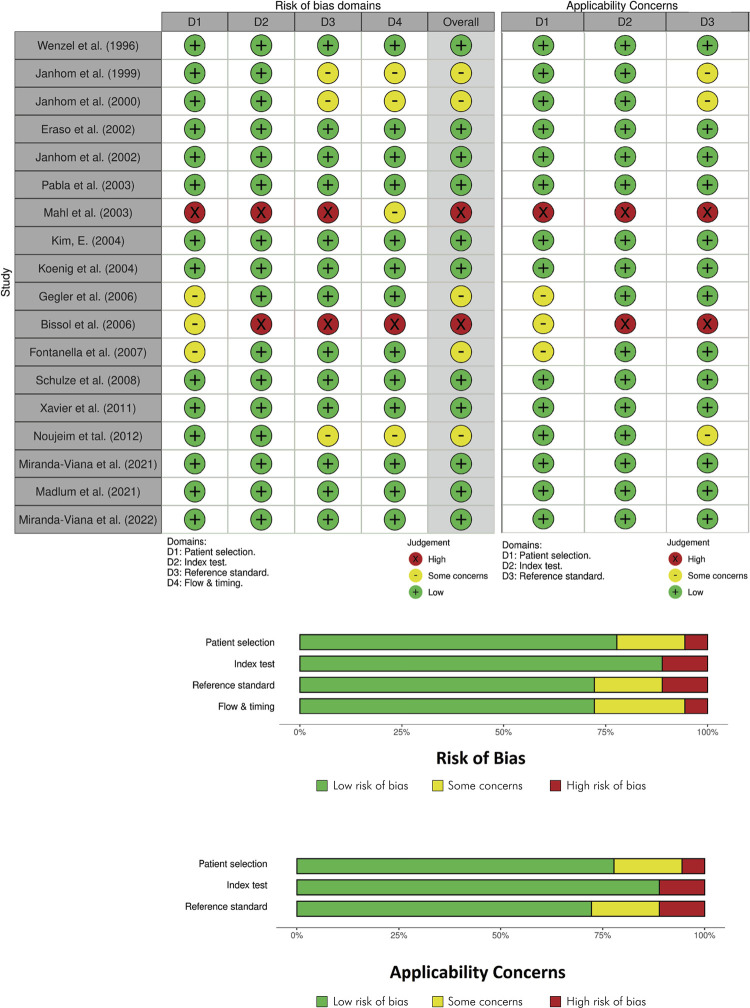
Flowchart and summary plot of the risk of bias and applicability concerns of the included studies

Figure 6 presents the flowchart and summary plot of the risk of bias and applicability concerns of the included studies. Regarding patient selection, most of the articles showed a low risk of bias due to the precise sample selection and standardization. Only four studies presented a medium to high risk of bias, because of failure to report randomization and inadequate sample exclusions.^10,13,14,19^ Regarding the index test and reference standard, a low risk of bias prevailed in most of the selected articles. Only five studies were scored with ’some concerns’^5,6,18^ and ‘high risk of bias,’^10,19^ since it was not clear whether the evaluators of the radiographic images were blinded regarding the factors studied, or whether they used a five-point scale to score the images assessed. Moreover, the lower number of evaluators was another factor that downgraded the score in this domain.^19^ Likewise, in the fourth domain, most of the selected articles also had a low risk of bias. The same number of studies (n = 5) were scored with ‘some concerns’^5,6,10,18^ and ‘high risk of bias,’^19^ because the information provided about the radiographic evaluation was not clear, and because it was not known whether these evaluations were compared with a reference standard according to each diagnostic task evaluated. Regarding the applicability domains, similar results were achieved within three domains (patient selection, index test, and reference standard), as presented earlier. Thus, based on the sum of the results, the overall score indicated ‘low risk of bias’ in 11 studies,^2-4,7-9,11,12,15-17^ suggesting accurate, standardized, and reliable results.

## Discussion

A scoping review aims to explore, evaluate, and map the literature. Thus, this scoping review was conducted to report and summarize the influence of image file formats on dental diagnostic tasks, according to assessments of intraoral radiographic images, since the studies presented divergent results. In addition, it is important to analyze the methodological quality of the studies, clarify the divergences among the concepts, and establish a consensus on the aspect of diagnostic accuracy. Eighteen studies were included in this scoping review, and the different image file formats in most of them did not influence the diagnostic accuracy in different dental diagnostic tasks. In addition, more than 70% of the included studies showed an overall score of ‘low risk of bias.’

The first study investigating the influence of image file formats on a dental diagnostic task was published in 1996.^4^ The study evaluated different compression ratios of the JPEG file format on the assessment of caries lesions. A multitude of studies have emerged from this first publication, addressing the impact of diverse file formats on dental diagnostic tasks.^2,3,4-12,13-19^ Of the 18 articles included in the current scoping review, ten^2,4-12^ assessed the influence of the image file formats on caries lesions detection. The high prevalence of this disease is explained by its being the most commonly investigated diagnostic task.^25^ With the exception of the study by Bissol et al.,^10^ which scored this disease as ‘high risk of bias,’ the other studies had scores from ‘moderate to low risk of bias.’^2,4-9,11,12^Among the studies presenting ‘low risk of bias,’^2,4,7-9,11,12^ four showed no significant influence of the file format on caries lesion diagnosis.^2,9,11,12^ All these studies were fundamental for understanding the different possibilities of exporting radiographic images, and for showing how radiographic images can be used in different file formats that reduce their size, such as the JPEG format, without impairing radiographic quality and diagnosis.^2,3,15,18^ A positive and relevant advantage for oral radiology clinics is being able to avoid the waste of virtual space, and allow the transfer of files more quickly between clinics and professionals.^1,2^

Interestingly, the current scoping review revealed that there was a lack of publications on this subject between 2013 and 2020. This lack may be attributed to developed countries’ having easier access to large storage drives and cloud-based tools, thus reducing the need for exporting radiographic images at a high compression level. This hypothesis is underpinned by the fact that half of the studies were from South America (nine studies - 50%). However, despite the controversial issues of the previous studies, such as unclear results, disproportional inclusion of image file formats (studies that evaluated a single format),^14,16,19^ and discrepancies between the compression ratios applied in the trials,^5,4,17^ three articles were recently published between 2021 and 2022.^2,3,15^ Overall, these studies assessed caries lesions,^2^ root resorptions,^3^ and root fractures,^15^ and no significant differences were found in the diagnosis among the different image file formats.

All the studies included evaluated the JPEG file format. The motivation for studying this format may be related to the small file size, in comparison with TIFF, PNG, BMP, and DICOM. The studies hypothesized that the diagnostic accuracy of JPEG could be negatively affected by the smaller size of its radiographic images. In disagreement with this hypothesis, most studies showed that the accuracy was not affected regardless of the image file format chosen or the diagnostic assessed. Thus, the clinicians can use the smallest file size option to satisfy the need for less virtual space and easier transmission of files between professionals.^2,3,8,11-15,18^ In addition to the aforementioned file formats, three studies evaluated a specific file format named JPEG2000 or wavelet format.^7,9,11^ Although this file format had been previously investigated by studies published from 2002 to 2008, its current use is uncommon, since it is supported by only some discontinued radiographic systems, and has low global reach, compared to the JPEG file format.^26^

Another interesting result is that none of the studies tested the DICOM file format. The DICOM file format was developed to standardize digital imaging and communication in medicine. Several countries, especially those in North America and Europe, already use this file format to transmit two-dimensional and three-dimensional images.^27^ However, unlike file formats with three-dimensional images, the DICOM file format for radiographic images is not recognized by the graphic system of these formats to allow immediate image viewing. Thus, to visualize a radiographic image in DICOM format, this image must be exported to specific viewer software, but this action hampers the process of evaluation and transmission of radiographic images, in comparison with the other file formats. The need for intermediate viewer software may explain why the included studies did not investigate the DICOM file format to perform radiographic evaluations of dental diagnostic tasks. Some studies that used the DICOM format were encountered in the selection process. However, these articles were not included because of the specific characteristics of these studies: literature review studies, such as Burgess’s study (2015)^27^; studies focused on images from a different body region, like femur fractures, such as the study by Botser et al.;^28^ studies that did not compare image file formats, but focused solely on DICOM image visualization, without considering the impact of the format itself on diagnostic accuracy, such as the studies by Gakenheimer et al.^29^ and Kallio-Pulkkinen et al.;^30^ and studies that did not apply the DICOM file format to a dental diagnostic task, such as the Kallio-Pulkkinen et al.^31^ and D’Addazio et al.^32^ studies. Therefore, it would be advisable to conduct future studies to assess the performance of the DICOM file format in subjective evaluations of different dental diagnostic tasks.

Summarizing the main results of the included studies, eight studies showed a significant influence of the file formats on diagnostic accuracy.^4-6,9,10,16,17,19^ Conversely, ten studies showed no significant effect of the file formats on the assessed diagnostic tasks.^2,3,7,8,11-15,18^ The compression ratios ranged from 1:1 to 1:47 among the studies that showed a significant influence of image file formats on diagnostic tasks, and from 1:1 to 1:26 among the studies that did not. Most studies that showed a significant influence of image file format on diagnostic accuracy used JPEG format with higher compression ratios, which is not clinically applicable because of the huge amount of loss of graphical information from the image, hence impairing the diagnosis. All the studies that showed no significant difference used the maximum compression ratio of 1:26, which is acceptable, because it does not impair the quality of the radiographic image. Contemporary radiographic systems support this compression rate, thus allowing exportation in JPEG format without affecting the memory space of the device, and facilitating the transmission of radiographic exams among professionals.^1,33^

Regarding the risk of bias in the studies included, nearly 70% received a ‘low risk of bias’ score across all four assessment domains (patient selection, index test, reference standard, and flow and timing). In the patient selection domain, three studies^10,13,14^raised ‘some concerns,’ and one had a ‘high risk of bias.’^19^ While most studies demonstrated standardized selection and randomization procedures with no inappropriate exclusions of radiographic images, those with bias concerns failed to clarify their sample selection and randomization processes,^13,14^ and excluded radiographic images without adequate explanation.^10,19^ Similarly, in the index test and the reference standard domains, two studies received a ’high risk of bias‘ score due to lack of clarity regarding the reference standard used to evaluate diagnostic accuracy.^10,19^ Three studies were assessed with an intermediate risk of bias, either due to an unclear description of the gold standard, or a fewer number of examiners assessing radiographic images than what is recommended.^5,6,18^ The importance of establishing a reference standard for diagnostic studies, and the potential impact of a low number of examiners on study results were emphasized in the respective studies. In the flow and timing domain, four studies raised ‘some concerns,’^5,6,18,19^ and one had a ‘high risk of bias’^10^ for insufficiently detailing whether all the images acquired were evaluated, and whether there was a timeframe between the evaluations to assess reproducibility. The predominant overall bias score among the included studies was ‘low risk’ in 11 out of 18 studies.^2-4,7-9,11,12,15-17^ Notably, seven studies with a low risk of bias found no impact of image file formats on diagnostic performance in dental tasks.^2,3,7,8,11,12,15^ It is crucial to underscore that the four studies identifying a significant effect used clinically unfeasible high compression rates.^4,9,16,17^ Thus, the current scoping review results support that any file format is applicable for radiographic diagnosis in dental diagnostic tasks, such as caries lesions, root resorptions, root fractures, periodontal disease, and periapical lesions.

In addition to methodological diversity and discrepancies in the findings of the studies, there was a significant limitation for conducting a meta-analysis in the present research, because of the lack of studies providing data on diagnostic accuracy, including sensitivity, specificity, positive and negative predictive values, and odds ratios when evaluating different image file formats in the diagnostic tasks investigated in each study. Although scoping reviews do not strictly require conducting a meta-analysis, similar to the assessment of bias in the included studies, it is crucial to acknowledge that comparative studies analyzing detection capability and agreement between imaging tests play an essential role in synthesizing underexplored scientific evidence.

Considering this aspect, it is pertinent to advise professionals who have challenges regarding the digital storage space of radiographic images to use the JPEG file format, since it has the smallest file size. Although the use of this file format is recommended, it should not be excessively compressed to ensure that the diagnosis is not impaired. However, it is important to underscore that local regulations should be considered before initiating clinical application. Moreover, there is a lack of knowledge of the radiographic image quality of DICOM files compared to the other formats. Therefore, future studies are encouraged to investigate the image quality of DICOM radiographic images compared to other formats.

## Conclusions

Based on the information extracted from the studies included in this scoping review, the most commonly applied radiographic images were periapical, and the most frequently studied diagnosis was that of caries lesions. The most widely used sensors were the direct digital ones, and the most frequently investigated formats were JPEG and TIFF files. Moreover, most studies concluded that there was no significant influence of different image file formats on diagnostic accuracy.

The current evidence of the influence of image file formats on dental radiographic diagnosis is reliable. Any image file format can be used, including those that demand greater compression ratios, without impairing diagnostic accuracy. Further primary studies using the DICOM file format are encouraged. Importantly, local regulations should be considered before clinical application.
